# Diversity, evolution and medical applications of insect antimicrobial peptides

**DOI:** 10.1098/rstb.2015.0290

**Published:** 2016-05-26

**Authors:** Eleftherios Mylonakis, Lars Podsiadlowski, Maged Muhammed, Andreas Vilcinskas

**Affiliations:** 1 Division of Infectious Disease, Warren Alpert Medical School of Brown University, Rhode Island Hospital, Providence, RI, USA; 2 Institute of Evolutionary Biology and Zooecology, University of Bonn, Bonn, Germany; 3 Institute for Insect Biotechnology, Justus-Liebig-University of Giessen, Giessen, Germany; 4 Department of Bioresources, Fraunhofer Institute for Molecular Biology and Applied Ecology, Giessen, Germany

**Keywords:** antimicrobial peptides, anti-infective, drug development, evolution, immunity, insects

## Abstract

Antimicrobial peptides (AMPs) are short proteins with antimicrobial activity. A large portion of known AMPs originate from insects, and the number and diversity of these molecules in different species varies considerably. Insect AMPs represent a potential source of alternative antibiotics to address the limitation of current antibiotics, which has been caused by the emergence and spread of multidrug-resistant pathogens. To get more insight into AMPs, we investigated the diversity and evolution of insect AMPs by mapping their phylogenetic distribution, allowing us to predict the evolutionary origins of selected AMP families and to identify evolutionarily conserved and taxon-specific families. Furthermore, we highlight the use of the nematode *Caenorhabditis elegans* as a whole-animal model in high-throughput screening methods to identify AMPs with efficacy against human pathogens, including *Acinetobacter baumanii* and methicillin-resistant *Staphylococcus aureus*. We also discuss the potential medical applications of AMPs, including their use as alternatives for conventional antibiotics in ectopic therapies, their combined use with antibiotics to restore the susceptibility of multidrug-resistant pathogens, and their use as templates for the rational design of peptidomimetic drugs that overcome the disadvantages of therapeutic peptides.

The article is part of the themed issue ‘Evolutionary ecology of arthropod antimicrobial peptides’.

## 1. Introduction

Antimicrobial peptides (AMPs) are short immunity-related proteins that can act against bacteria, viruses, fungi or parasites. In insects, they are secreted from cells and tissues that contribute to host innate immunity such as the haemocytes or the fat body and they could be a valuable alternative to conventional antibiotics in the era of growing antimicrobial resistance [1–3]. AMPs participate in several defence-related processes, including the killing of pathogens, the ability to bind and neutralize endotoxins and to modulate the immune responses to infection [4]. The induction of AMPs in insects mediates a temporary humoral immune response characterized, for example, by enhanced AMP concentrations in the haemolymph, which is longer lasting than the initial cellular responses and which is believed to function as a back-up against persistent infections [5]. The functions of AMPs have been expanded beyond their role in defence against pathogens to include also the control of endosymbionts [6]. AMPs are ubiquitous among eukaryotes, but have been much more intensively studied in insects [7,8]. The non-ribosomally AMPs produced by bacteria and fungi are different from those contributing to the innate immunity of multicellular organisms which are ribosomally produced, and the term AMP usually refers specifically to these molecules [9]. A comprehensive list of known AMPs can be found in the Antimicrobial Peptide Database (APD) (http://aps.unmc.edu/AP).

In this work, we review the diversity of insect-derived AMPs, and evolution of insect AMPs by mapping their phylogenetic distribution. We highlight the use of the nematode *Caenorhabditis elegans* as a whole-animal model in high-throughput screening methods to identify AMPs with efficacy against human pathogens. We also discuss the potential medical applications of AMPs, emphasizing their roles as antimicrobials.

## 2. Insect antimicrobial peptides

### (a) Classification of insect antimicrobial peptides

Insects produce a larger repertoire of AMPs than any other taxonomic group, and the number of individual AMPs produced by each species varies considerably. At one end of the scale, the invasive ladybird *Harmonia axyridis* is known to produce more than 50 AMPs [10]. At the other end, the pea aphid *Acyrthosiphon pisum* does not produce any known AMPs that act against bacteria [11]. The AMP repertoire is conceivably linked to the nature of the environmental threats faced by each species during evolution, i.e. those exposed to more pathogens, and more diverse pathogen species, would be expected to have evolved a broader repertoire of AMPs [12,13]. Insect AMPs display a remarkable evolutionary plasticity in terms of gain, loss and functional shifts of the coding genes. The last encompasses duplication and divergent evolution of AMPs, which can ultimately result in new functions of the resulting paralogues. This neo-functionalization enables adaptation to emerging pathogens, but also switches between immunity and non-immunity-related functions [14,15].

The increasing number of published insect genome and transcriptome datasets combined with the ability to probe haemolymph samples directly using proteomics techniques has resulted in the discovery of many new AMPs in the past few years [12]. Novel AMPs can be identified by homology to known peptides, but also by other features such as the presence of protease cleavage sites that release the mature peptide from propeptide precursors, and expression profiles that focus on immunocompetent cells and tissues such as haemocytes and the fat body [16,17].

Insect AMPs can be classified according to their structure or function. The three major structural classes are linear α-helical peptides without cysteine residues, peptides with a β-sheet globular structure stabilized by intramolecular disulfide bridges, and peptides that contain unusually high numbers of specific amino acid residues, such as proline or glycine [1,7,9]. Where secondary structures and disulfide bridges are present, these elements are often necessary for AMP activity [1,3,4,8]. The functional categorization of insect AMPs tends to be based on target pathogen range rather than any specific mechanism of action. Some have a broad range, whereas others show varying degrees of specificity towards Gram-positive or Gram-negative bacteria, fungi, parasites and even viruses [17,18].

The first insect AMP to be discovered was cecropin, so-called, because it is produced by larvae of the giant silk moth *Hyalophora cecropia*. This is the prototype α-helical linear AMP, and it is active against Gram-negative bacteria such as *Escherichia coli* [19,20]. Other cecropins have been identified more recently, as well as additional cecropin-like peptides such as sarcotoxins, hyphancin and enbocin, which can act against both Gram-positive and Gram-negative bacteria [21]. Most cecropins contain a tryptophan residue at or near the N-terminus, a long N-terminal amphiphilic *α*-helix, a shorter and more hydrophobic α-helix at the C-terminus and an amidated C-terminal residue.

Defensins represent the prototype for the second major structural class of insect AMPs [22]. They have a predominantly β-sheet globular structure, they are stabilized by intramolecular disulfide bridges [22], and they are widely distributed among different insect orders including ancient apterygote insects [23], hemimetabolous orders such as Hemiptera and Odonata [24], and holometabolous orders such as Coleoptera, Diptera, Hymenoptera and Lepidoptera [10,13,16,21,25]. The first insect peptides described as defensins were discovered in the flesh fly *Phormia terranovae* and were found to be active against Gram-positive bacteria [26], although similar peptides had already been identified in *Sarcophaga peregrine* [27]. Most insect defensins act against Gram-positive bacteria, although some also inhibit Gram-negative bacteria [10,21,25,26]. A small number of insect defensins act exclusively against filamentous fungi, e.g. gallerimycin from the greater wax moth *Galleria mellonella* [28].

A key example of the third structural group of AMPs is the proline-rich AMPs. As well as the characteristic multiple proline residues, these AMPs are 15–39 residues in length and feature two domains, one of which is highly conserved and confers general antimicrobial activity, whereas the other is more variable that mainly confers target specificity [29]. Proline-rich AMPs have been identified in the Diptera (drosocin and metchnikowin) [30], Hemiptera (pyrrhocoricin and metalnikowins) [31], Hymenoptera (apidaecins, abaecins and formaecins) [32,33] and Lepidoptera (lebocins) [34]. The proline-rich AMPs can be further divided into short-chain (20 residues or fewer) and long-chain (more than 20 residues) subfamilies, the former showing more potent activity against Gram-negative bacteria, whereas the latter are more active against Gram-positive bacteria and fungi [35,36]. Such differences in their specificity may be mediated by their distinct lipopolysaccharide binding activity or their ability to penetrate bacterial membranes, because at least some proline-rich AMPs are known to target intracellular molecules [31,34].

Another example of the third structural group is the glycine-rich AMPs. These peptides have been identified among the Coleoptera (coleoptericin, holotricin 2, holotricin 3, tenecin 3 and acaloleptin A), Diptera (diptericins, attacins and sarcotoxin II), Hemiptera (hemiptericin), Hymenoptera (hymenoptaecins) and Lepidoptera (attacins and gloverins) [7,10,13,17,37]. Most glycine-rich AMPs are highly specific for particular groups of Gram-negative bacteria, although honeybee hymenoptaecin also shows activity against the Gram-positive species *Micrococcus lysodeikticus* and *Bacillus megaterium* [38]. Our knowledge explaining the specificity of individual glycine-rich AMPs is fragmentary, but studies with attacins and gloverins from Lepidoptera implicate the presence of different targets. Attacin from *Hylophora cecropia* was found to inhibit outer membrane synthesis in *E. coli* [39,40], whereas gloverin from *Manduca sexta* binds to Gram-positive bacterial lipoteichoic acid, peptidoglycan and different moieties of lipopolysaccharide [41].

### (b) Modes of action of insect antimicrobial peptides

Most insect AMPs have a net positive charge and contain up to 50% hydrophobic residues [1,7,9]. This leads to interaction of those AMPs with the negatively charged and lipophilic membranes of bacterial cells, reflecting the abundance of acidic phospholipids in the outer leaflet, in contrast to the membranes of eukaryotic cells which are dominated by zwitterionic and uncharged lipids [42]. AMPs are therefore electrostatically attracted to bacterial cell membranes, and once contact is established the hydrophobic residues promote integration, causing the outer leaflet of the membrane to expand and become thinner, ultimately creating pores or even causing lysis.

The ability of AMPs to increase membrane permeability has been confirmed in experiments involving dye-loaded bacteria exposed to *Papilio xuthus* cecropin A and papiliocin, resulting in extensive dye leakage [43]. Models that offer mechanistic explanations for the interaction between AMPs and bacterial membranes have been reviewed extensively elsewhere [4,9]. Although such models are attractive in their simplicity, they cannot yet explain how AMPs overcome barriers such as the peptidoglycan-rich bacterial cell wall [4,44]. The binding of AMPs to anionic lipopolysaccharides or teichoic acids appears essential for their activity, because charge neutralization makes bacteria more resistant to cationic AMPs [39–41,44]. Although most AMPs appear to function by increasing membrane porosity, the proline-rich AMPs (such as bumblebee abaecin) are instead thought to interact with intracellular targets representing the bacterial chaperone network such as DnaK or the protein synthesis apparatus [45]. For example, attacins have been shown to inhibit the synthesis of proteins which are components of the outer bacterial membrane [39,40]. Furthermore, some AMPs have been shown to inhibit cell wall synthesis by interfering with the corresponding enzymes or lipid phosphatidylethanolamines, or by delocalization of bacterial cell surface proteins [41]. Other insect AMPs such as the insect metalloprotease inhibitor (IMPI) neutralize specifically virulence-associated microbial metalloproteases [46].

An emerging aspect is that some co-occurring AMPs, which are, for example, simultaneously induced during immune responses, can enhance or enable the activity of others [45]. Besides such potentiation there are also examples for synergistic activity of AMPs. For example, a defensin (LSer-Def4) and a cecropin (LSer-Cec6) from the wound maggot *Lucilia sericata* display greater than additive antibacterial activity when tested in combination [21]. In agreement, synthesized bumblebee AMPs have recently been shown to display combinatorial activity against parasites such as the trypanosome *Crithidia bombi* [47].

## 3. Phylogeny of hexapod/insects and the evolution as well as distribution of antimicrobial peptides

**Figure 1.**
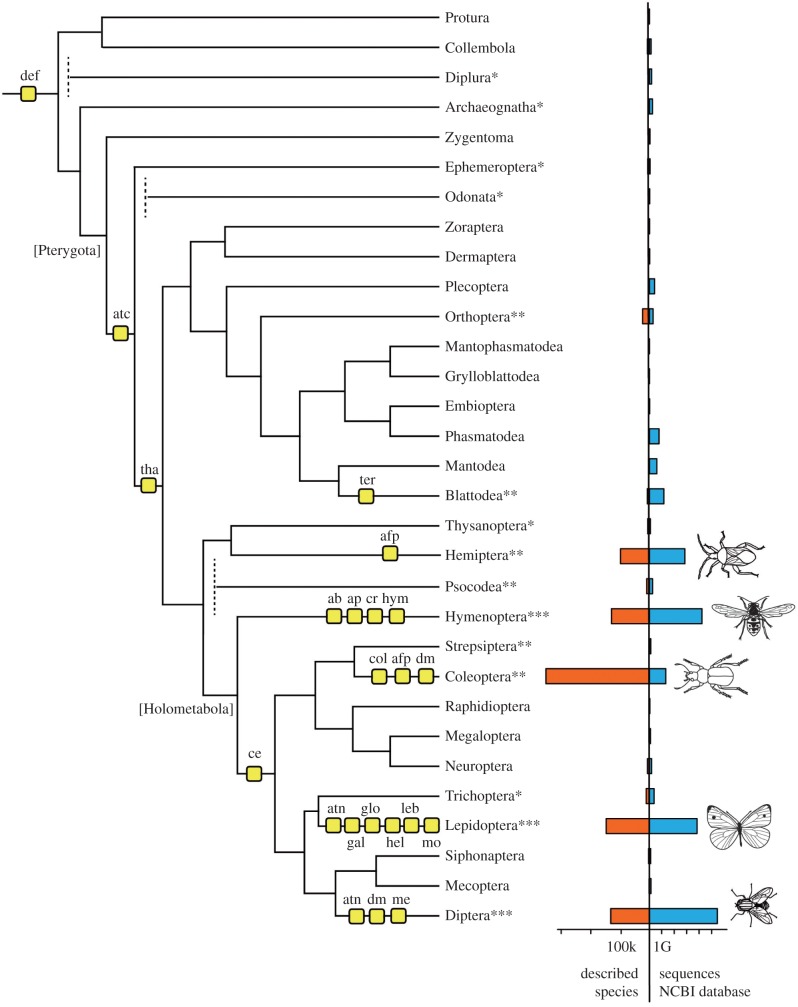
Evolution of AMPs in insects. The topology largely follows a recent analysis of more than 150 species and concatenated alignments of 1478 genes derived from transcriptomic data [48]. Remaining controversies are depicted by dashed branches (Diplura, Odonata, Psocodea). Following modern taxonomy the Isoptera (termites) are included as a subclade of Blattodea, and Phthiraptera (true lice) are nested inside Psocodea. Asterisks mark taxa with genome projects (one asterisk: genome data available in database of i5k pilot project, but not yet officially published; two asterisks: one to three published genome projects; three asterisks: more than three published genome projects). The barplot shows the number of described species (orange bars to the left) and the number of publicly available nucleotide sequences (blue, to the right) as a measure for intensity of research (combined number of NCBI nucleotide and EST databases; evaluated on 14 October 2015). Hypothesized evolutionary origins of AMP families are mapped on the tree. Note that due to the incomplete representation of immune challenged transcriptomes or genomic data from insect orders, many AMPs may also have evolved on earlier branches of the tree. Abbreviations: ab, abaecin; afp, antifungal protein; ap; apidaecin; atc, attacin C-terminal domain; atn, attacin N-terminal domain; ce, cecropin; col, coleoptericin; cr, crustin; def, defensin; dm, drosomycin; gal, gallerimycin; glov, gloverin; hel, heliomicin; hym, hymenoptaecin; leb, lebocin; mor, moricin; ter, termicin; tha, thaumatin.

Study of evolution of novel genes participating in insect antimicrobial defence is a very interesting field. Hexapod AMPs may arise by gene duplication and subsequent diversification, by horizontal gene transfer or by *de novo* creation from non-coding sequences. A well-resolved, dated phylogeny of insects is available [48], providing an indispensable resource that can be used to analyse the evolutionary history of gene families including AMPs. However, the emergence of AMP genes mapped onto the insect phylogenetic tree (figure 1) shows [48] that most of the known AMPs are found in insect taxa with completed genome projects. This is not surprising because model insects with a completely sequenced genome are most intensively studied for a variety of biological questions, including AMPs and immunity. This suggests there is significant undiscovered AMP diversity in insects hidden among the less well-characterized insect taxa and the underrepresented families of the megadiverse orders. AMPs can arise *de novo* in restricted phylogenetic lineages over very short evolutionary timescales. Furthermore, tracing the evolution of AMPs in parallel with hexapod phylogeny involves two major obstacles. First, there is a substantial bias in favour of the insect taxa with abundant sequence data and against branches that are particularly underrepresented (figure 1). Most insect AMP genes have been discovered in the five megadiverse orders (Coleoptera, Diptera, Hemiptera, Hymenoptera and Lepidoptera), which encompass more than 90% of known insect species, 77% of the nucleotide sequences in the NCBI genomic and expressed sequence tag (EST) databases, and more than 95% of predicted protein sequences. Nevertheless, these megadiverse orders comprise only a part of hexapod diversity. They represent only the crown of the hexapod tree, spanning about 370 Myr of insect evolution, whereas the base of the tree is probably more than 100 Myr older and several early branching lineages remain to be studied in detail [48].

The second major obstacle is the recognition of homology among AMP genes. Their short length, substantial variation between species, as well as frequent gain and loss during evolution [12,14,15], hamper the identification of orthologues and often prevents the recognition and delimitation of AMP families. For example, the assignment of novel glycine-rich, proline-rich, defensin-like or cecropin-like peptides is often frustrated by the unconvincing homology revealed by sequence alignments or hidden Markov models (HMMs), as discussed in detail for the attacins in §3a. More groundwork is therefore required for the classification and delimitation of AMP families, and herein we therefore present only a brief overview of the evolutionary history of selected insect AMPs. We define AMP families by significant (best) hits with HMMER v. 3.1 [49] against domains listed in the PFAM protein family database (http://pfam.xfam.org). In those cases where PFAM cannot provide an HMM model, we define HMMs based on sequence alignments (e.g. lebocin and hymenoptaecin) or individual sequences (e.g. gallerimycin).

### (a) Antimicrobial peptides that are widely distributed among insect taxa

Important examples of widely distributed AMPs derived from insects are defensin (will be discussed in §3c), cecropins and attacins. Cecropins (PFAM: PF00272) were first discovered in the Lepidoptera [50] and are found in the orders Coleoptera, Diptera and Lepidoptera, but not Hymenoptera. Given that Hymenoptera is a sister order for the other Holometabola, we predict that cecropins have probably evolved once (figure 1) and may be present in the Holometabola orders that remain to be characterized. Some AMPs found beyond the Holometabola have also been described as cecropins, but these share only superficial similarity with the cecropin domain defined by PFAM.

The attacins are represented by discrete N-terminal and C-terminal domains (PFAM: Attacin_N, PF03768; Attacin_C, PF03769). Full-length attacins containing both domains were first described in the Lepidoptera and other attacins were later found in certain brachyceran Diptera [51,52], but not in mosquitoes or chironomids. The alignment of dipteran and lepidopteran attacin N-terminal domains reveals no similarity, suggesting the HMM for Attacin_N is artificial. Furthermore, we have mined the transcriptomes of five Trichoptera species, four Mecoptera species and three Siphonaptera species without finding an attacin N-terminal domain (L. Podsiadlowski 2015, unpublished data). Given the distant phylogenetic relationship between Lepidoptera and Diptera–Brachycera, these N-terminal domains are likely to have evolved independently in the two taxa. By contrast, the C-terminal attacin domain is present in most of the Holometabola genomes studied thus far, and also in the orders Orthoptera (Locusta), Isoptera (Macrotermes) and Hemiptera (Rhodnius). In the latter case, the AMP was named prolixicin [53]. The diptericins [54] also show similarities with the Attacin_C domain, suggesting that an AMP resembling the attacin C-terminal domain was present at the base of the clade known as Neoptera (figure 1). We thus propose that a novel attacin consisting of N- and C-terminal domains evolved independently in the lineages Brachycera and Lepidoptera. This is in line with the hypothesis that the genes encoding three attacins and diptericin from *Drosophila* share a common ancestor [55].

### (b) Antimicrobial peptides restricted to individual insect orders

Several AMP families have only been identified in a single insect order or even in a more restricted taxonomic group. For example, moricin (PFAM: PF06451) [56], glycine-rich gloverin (PFAM: PF10793), proline-rich lebocins [57] and the antifungal cysteine-rich peptides heliomicin (with similarity to PFAM toxin_3 domain, PF00537) [58] and gallerimycin [59] have only been found in the Lepidoptera.

Metchnikowin (PFAM: Antimicrobial10, PF08105) is a proline-rich AMP only found in the genus *Drosophila* [60], and it has been identified in all 12 *Drosophila* genomes sequenced thus far [61]. The coleoptericins (PF06286) are glycine- and proline-rich peptides which, as the name suggests, are only found in the Coleoptera. The first coleoptericin was discovered in larvae of the tenebrionid beetle *Zophobas atratus* [60], followed by similar peptides in other beetles such as *Tribolium castaneum* [62], the harlequin ladybird *H. axyridis* [10] and the longicorn beetle *Acalolepta luxuriosa* [37]. The latter has a remarkable coleoptericin gene comprising a multi-peptide precursor, which yields up to five mature peptides. Termicin (PFAM domain: Toxin_37, PF11415) is a knottin-type AMP discovered in termites [63], which is also found in their closest relatives, the cockroaches (e.g. *Periplaneta americana* EST library, NCBI FG130406, and *Eupolyphaga sinensis* cDNA, NCBI KR014250).

Several AMPs are also thought to be restricted to the Hymenoptera, including the proline-rich peptide abaecin (PFAM: Antimicrobial_5, PF08026) which is found in bees [64], ants [32,33,65], the genus *Nasonia* [66] and another pteromalid wasp [67]. Apidaecin (PF00807) is only found in bees (the genera *Apis*, *Bombus*, *Megachila* and *Melipona*) and may therefore represent a recent evolutionary adaptation [68]. Finally, hymenoptaecin [38] is a glycine-rich peptide, found only in bees, ants, and wasps from the genus *Nasonia* [66].

Crustins (containing a PFAM WAP-domain, PF00095) were discovered in crustaceans, but similar sequences have been identified in hymenopteran genomes [66]. They probably have a wider distribution among hexapods, e.g. we found short peptides with WAP domains in the genomes of the coleopteran *T. castaneum* (TC11324) and two termite species (*Zootermopsis nevadensis* ZNEV05303 [69] and *Macrotermes natalensis* MNAT10208).

### (c) Antimicrobial peptides with a scattered distribution over unrelated taxa

The arthropod defensin family (PFAM: Defensin_2, PF01097) is the only hexapod AMP family that is broadly distributed beyond the insects, e.g. in ticks and scorpions, where modified defensins are a component of the toxin blend [70]. Other ‘defensins' are found in diverse invertebrates (e.g. molluscs and roundworms), as well as in vertebrate and plant species, but their sequence diversity and small size make it difficult to confirm a unique evolutionary origin. Arthropod defensins are found in diverse hexapod species, including the orders Zygentoma [23] and Odonata [24], clearly suggesting that an arthropod defensin was already present in the last common ancestor of all insects.

Drosomycin (PFAM: Gamma-thionin PF00304) was first identified in *Drosophila melanogaster* [71], but it is not present in all *Drosophila* species. Among the Diptera, this AMP is also found in *Musca domestica* but not in *Glossina*, *Phlebotomus* or any mosquito genome. Recently, a similar peptide was also discovered in two genera of beetles (*Callosobruchus* and *Trox*) [72], and there are also similarities with the cremycin family of nematode AMPs. The hypothesis that insect drosomycins and nematode cremycins may have been acquired from plants by horizontal gene transfer is supported by the patchy distribution pattern of homologues which are present in Ecdysozoa, absent in other animals, fungi and protozoa, but widespread in plants. The alternative explanation postulates the evolution of these antifungal peptides in the last common ancestor of all eukaryotes and independent loss in fungi and all Metazoa except Ecdysozoa [72].

Alo-3 (PFAM domain: PF11410) is a knottin-type antifungal peptide which was first identified in the beetle *Acrocinus longimanus* [73]. A related insect peptide was identified in the whitefly *Bemisia tabaci* (ABC40569-ABC40572). Both species are phytophagous. Because this domain is otherwise only found in plants and fungi, it may be another example of horizontal gene transfer from plants, and at least two independent events are likely to have occurred given the phylogenetic distance between Coleoptera and Hemiptera (figure 1).

Thaumatins are antifungal peptides (PFAM domain: PF00314) which have been identified in fungi, plants and animals, and in the last case they appear to be restricted to nematodes, ticks and insects. In a broad comparison of all eukaryotic thaumatins, all animal thaumatins form one clade, nested within plant thaumatins [74]. This suggests that animal thaumatins have a single origin, perhaps reflecting a horizontal gene transfer event early in the evolution of the Ecdysozoa (a clade including nematodes and arthropods). Thaumatins are found in many but not all insect orders. They are present in beetles [75], aphids [11] and termites [69], but among the Diptera, thaumatin has been identified in two chironomids (*Polypedilum vanderplancki* PVAN02763 and *Polypedilum nubifer* PNUB14148), but in none of the 32 complete genomes representing mosquitoes and flies. Thaumatin proteins are also scarce among the Lepidoptera and Hymenoptera, despite the availability of several genome datasets. Although the scattered distribution suggests independent acquisition events, the fact that all arthropod thaumatins are more closely related to each other than to fungal or plant thaumatins seems to rule out this hypothesis and favour multiple independent losses of thaumatin genes in several hexapod lineages. Despite the number of thaumatin encoding sequences in insects, only a thaumatin gene identified in *T. castaneum* has been heterologously expressed and confirmed to display antifungal activity [75].

## 4. Activity screening and medical applications of insect antimicrobial peptides

The number of infections caused by drug-resistant microbes is increasing, particularly those involving ‘ESKAPE’ bacteria (*Enterococcus faecium, Staphylococcus aureus, Klebsiella pneumoniae, Acinetobacter baumanii, Pseudomonas aeruginosa, Escherichia coli* and *Enterobacter* species) that ‘escape’ the effects of current antimicrobial drugs, pose a substantial threat to public health, and contribute significantly to patient morbidity [76,77]. In addition to the ESKAPE bacteria, other pathogens including *Clostridium difficile*, *Candida* species [78] and multidrug-resistant/extensively drug-resistant mycobacteria provide further evidence that we are losing the war against emerging resistant microbes [76,77,79].

The Centers for Disease Control and Prevention (CDC) report that two million people per year in the USA acquire serious infections due to bacteria that are resistant to one or more current antibiotics (www.cdc.gov/). This results in 23 000 deaths as a direct result of these infections and many more due to complications, costing up to US$20 billion in excess direct healthcare costs and annual productivity losses exceeding US$35 billion. Similarly, the European Centre for Disease Prevention and Control (ECDC) claim that antibiotic resistance costs the European Union approximately €1.5 billion per year (ecdc.europa.eu/). Agencies such as the US President's Council of Advisors on Science and Technology (PCAST), have highlighted the need to focus on antibiotic drug discovery to address the issue of drug-resistant pathogens [80]. Accordingly, the UK has recently launched the Five Year Antimicrobial Resistance Strategy 2013–2018 (https://www.gov.uk/government/uploads/system/uploads/attachment_data/file/244058/20130902_UK_5_year_AMR_strategy.pdf), a collaboration involving multiple agencies including government departments and academic centres. Importantly, one of the successful approaches to identifying potential antimicrobials is the high-throughput screening method using the nematode *C. elegans* as alternative host. This method was further applied to identify AMPs as alternative antimicrobials.

### (a) *Caenorhabditis elegans* as a high-throughput screening model to identify antimicrobial peptides with activity against human pathogens

The identification of compounds with *in vivo* activity against human pathogens and their development as new drugs is challenging, but high-throughput screening provides a more efficient way to identify such compounds quickly. High-throughput screening was first applied to drug development in the 1980s and the same concept can be used today to screen libraries of AMPs [81,82].

The nematode *C. elegans* is a model organism with a completely sequenced genome [83,84]. It is a small, free-living, transparent worm, which is approximately 1 mm in length and comprises about 1000 cells. It has a lifespan of two to three weeks, and many thousands of individual worms can be propagated on plates containing nematode growth medium agar spiked with lawns of non-pathogenic bacteria [85]. *Caenorhabditis elegans* is widely used for the *in vivo* investigation of host–pathogen interactions, including the study of microbial pathogenesis and innate immune responses [86–88]. Notably, several evolutionarily conserved innate immune response mechanisms have been described in *C. elegans*, including p38 mitogen-activated protein kinase (MAPK) pathways [89,90]. Moreover, various antimicrobial agents have been shown to prolong the survival of infected worms, confirming that this model organism is suitable for antimicrobial discovery [87,91,92]. Furthermore, the *C. elegans–*microbe liquid assay allows the identification of agents that directly kill microbes or that possess immunomodulatory and anti-virulence effects. This host–pathogen system has been further developed to enable high-throughput experiments in an automated robotic system which is used to dispense medium, worms and compounds onto the assay plates, and which monitors nematode survival manually or by image-based automated screening [93,94].

The suitability of *C. elegans* for the high-throughput screening of antimicrobial compounds was first demonstrated using *Enterococcus faecalis* as a model pathogen [93]. More recently, high-throughput screens using *C. elegans* have identified antimicrobial agents that work against challenging pathogens such as methicillin-resistant *Staphylococcus aureus* (MRSA), *Pseudomonas aeruginosa* and *Candida albicans* [94,95]. Strikingly, using this assay in a screening of 68 synthetic insect-derived AMPs, we determined that defensin 1 from the red flour beetle *T. castaneum* displays synergistic activity with the antibiotics Telavancin and Daptomycin against multidrug-resistant *S. aureus* [96]. These results open a new avenue for the application of insect-derived AMPs. In combination with antibiotics they could be used to restore the susceptibility of multidrug-resistant pathogens.

We have established a *C. elegans*–*Acinetobacter baumannii* assay to conduct a pilot screen on a library of 68 insect-derived AMPs. This screen identified 15 cecropins and cecropin-like AMPs that prolonged the survival of *C. elegans* infected with *A. baumannii* [97]. One of the identified AMPs (BR003-cecropin A), isolated from the mosquito *Aedes aegypti* was found to be effective against multiple species of Gram-negative bacteria and to act with a low minimum inhibitory concentration against different *A. baumannii* strains [97].

### (b) Potential medical applications of antimicrobial peptides

Previous reports have highlighted several potential medical roles for AMPs [3,8,17,98,99]. As stated above, they have diverse mechanisms of action including the inhibition of gene expression or protein synthesis (e.g. targeting ribosomal proteins, RNA polymerase, or directly binding to DNA), the inhibition of cell wall synthesis (e.g. by targeting the corresponding enzymes or lipid phosphatidylethanolamines), or the delocalization of bacterial cell surface proteins [98,99]. Some AMPs have demonstrated immune-stimulatory effects by inducing cell migration, cell proliferation or the release of cytokines and chemokines [100]. Other insect-derived AMPs such as Harmoniasin from the harlequin ladybird *H. axyridis* may be useful leads for the development of anti-cancer drugs [101].

Indeed, there is an increasing number of insect-derived AMPs shown to inhibit human pathogens. Examples of susceptible bacteria include multidrug-resistant *A. baumannii*, *Bacillus coagulans*, *Citrobacter freundii*, *Enterobacter aerogenes*, *E. coli*, *Francisella tularensis*, *K. pneumonia*, *Legionella dumoffei*, *Listeria monocytogenes, Proteus vulgaris*, *S. aureus* and *Streptococcus sanguinis* [17,21,53,96,97,102,103]. Examples of fungi which are susceptible to insect AMPs include *Aspergillus fumigatus*, *Alternaria* spp., *Botrytis cinerea, C. albicans*, *Cryptococcus neoformans*, *Fusarium* spp., *Neurospora crassa*, *Pichia pastoris, Trichoderma viridae* [17,28,37,104]. Of note is that various insect AMPs have been shown to inhibit viruses including a mellitin derivative (hecate) from the honeybee (*Apis mellifera*) which is active against *Herpes simplex virus 1* (HSV-1) [100], and two alloferons from the blowfly *Calliphora vicina* which are active against *Human influenza* viruses A and B [105].

There is a debate about the ability of bacteria to evolve resistance against AMPs. On the one hand, many researchers argue that AMPs such as defensins retained their antibacterial activity over millions of years and only modest resistance development has been observed under *in vitro* selection pressure [106]. Further, exposure to AMPs elicits neither stress responses nor increased mutation rates in treated bacteria [107]. On the other hand, there are several mechanisms described which can mediate bacterial resistance against AMPs [108].

Bacteria-derived gramicidins represent the first peptide antibiotics to become commercially available [109] and numerous reports have highlighted that insect-derived AMPs are attractive candidates to be developed as alternatives to conventional antibiotics [3,8,17,98,99,106]. Despite worldwide efforts, there are no insect-derived AMPs on the market yet. Their development as antibiotics requires solutions to some obstacles which will be briefly addressed.

The testing of insect-derived AMPs displaying potent *in vivo* and *in vitro* activity in preclinical and clinical studies requires amounts which are difficult to produce economically [110]. However, the costs for the synthesis of short peptides (up to approx. 80 residues) have decreased markedly in recent years [111], enabling at least the production of insect-derived AMPs which are too small to be immunogenic or allergenic. For the larger insect AMPs, particularly those with complex three-dimensional structures that are stabilized by intramolecular disulfide bonds, we need cost-efficient heterologous expression systems, and insect cell lines in particular have proved to be promising tools for the production of functional insect-derived recombinant peptides [112]. The process development of cost-efficient insect cell-based protein production systems has become a major challenge in insect biotechnology [113] and the recently achieved solutions are groundbreaking, but still exceed the manufacturing costs of conventional drugs [114].

The bioavailability of drugs depends on their stability. The susceptibility of insect AMPs to host proteases differs markedly. Basically, linear peptides are generally more proteolytically degradable than those AMPs with an intramolecular structure stabilized by disulfide bonds such as that known from defensins [106]. The antibacterial activity of the latter hampers their heterologous production in bacteria which also usually lack the ability to synthesize properly folded functional peptides. These limitations can be overcome by the use of advanced insect cell-based expression systems [112–114]. The design of functional analogues or peptidomimetics which are more resistant to hydrolysis by host proteases, has emerged as another strategy to develop novel antibiotics using insect-derived AMPs as leads [115,116]. Further, only limited amounts of particular AMPs are required for systemic application if they are used to restore the susceptibility of pathogens to conventional antibiotics [117]. Consequently, the combinatorial use of insect-derived AMPs together with antibiotics has become another avenue of research aiming to implement these natural products in therapeutic approaches.

The above-mentioned obstacles for systemic application of insect-derived AMPs have favoured their development for ectopic applications which do require less demanding preclinical and clinical research [118]. A prime example is the development of AMPs from medicinal maggots of *L. sericata* which are formulated in hydrogels to test the efficacy of synthetic counterparts in wound dressings and as cosmetic ingredients to deter dermatological pathogens [21,104,119]. Other insect-derived AMPs such as the IMPI from *G. mellonella* [120] are currently also being developed for the therapy of chronic wounds [121]. Further promising medicinal applications of insect-derived AMPs are currently being explored in therapies to cure eye, lung and urogenital infections [117,122,123]. For example, it has been demonstrated that a defensin from *G. mellonella* or AMPs of medicinal maggots are active against causative agents of lung infection [21,117]. Recombinant analogues of insect-derived AMPs can be delivered to the lung bound on inhalable microparticles and the simultaneous application of AMPs displaying synergistic activity is expected to reduce the amounts required for therapeutic approaches.

## 5. Conclusion

An ever increasing number of AMPs are being found in insects. The corresponding genes display a remarkable evolutionary plasticity in terms of gain, loss and neo-functionalization. Mapping the presence of AMPs on the phylogenetic tree of insects reveals the existence of widespread and taxon-specific AMP families. Recent *in vitro* and *in vivo* screening using surrogate model hosts such as *C. elegans* has shown that insect-derived AMPs display promising activity against human pathogens that could make them suitable as alternatives to conventional antibiotics. However, their development must address the limitations associated with the application of peptide-based drugs.
